# Biological aging of CNS-resident cells alters the clinical course and immunopathology of autoimmune demyelinating disease

**DOI:** 10.1172/jci.insight.158153

**Published:** 2022-06-22

**Authors:** Jeffrey R. Atkinson, Andrew D. Jerome, Andrew R. Sas, Ashley Munie, Cankun Wang, Anjun Ma, William D. Arnold, Benjamin M. Segal

**Affiliations:** 1 Department of Neurology, College of Medicine, The Ohio State University Wexner Medical Center, Columbus, Ohio, USA.; 2 Neuroscience Research Institute, The Ohio State University, Columbus, Ohio, USA.; 3 Graduate Program in Immunology, University of Michigan Medical School, Ann Arbor, Michigan, USA.; 4 Department of Biomedical Informatics, College of Medicine, The Ohio State University, Columbus, Ohio, USA.

**Keywords:** Autoimmunity, Neuroscience, Autoimmune diseases, Multiple sclerosis, Neurological disorders

## Abstract

Biological aging is the strongest factor associated with the clinical phenotype of multiple sclerosis (MS). Relapsing-remitting MS typically presents in the third or fourth decade, whereas the mean age of presentation of progressive MS (PMS) is 45 years old. Here, we show that experimental autoimmune encephalomyelitis (EAE), induced by the adoptive transfer of encephalitogenic CD4^+^ Th17 cells, was more severe, and less likely to remit, in middle-aged compared with young adult mice. Donor T cells and neutrophils were more abundant, while B cells were relatively sparse, in CNS infiltrates of the older mice. Experiments with reciprocal bone marrow chimeras demonstrated that radio-resistant, nonhematopoietic cells played a dominant role in shaping age-dependent features of the neuroinflammatory response, as well as the clinical course, during EAE. Reminiscent of PMS, EAE in middle-aged adoptive transfer recipients was characterized by widespread microglial activation. Microglia from older mice expressed a distinctive transcriptomic profile suggestive of enhanced chemokine synthesis and antigen presentation. Collectively, our findings suggest that drugs that suppress microglial activation, and acquisition or expression of aging-associated properties, may be beneficial in the treatment of progressive forms of inflammatory demyelinating disease.

## Introduction

Traditionally, people with multiple sclerosis (MS) are categorized into 2 major clinical subsets based on whether they experience a relapsing-remitting or progressive course. Relapsing-remitting MS (RRMS) is characterized by discrete, self-limited episodes of neurological signs and symptoms that persist for weeks to months, followed by a partial or full recovery. Clinical relapses are associated with the appearance of acute inflammatory demyelinating lesions in CNS white matter, which are driven by the infiltration of lymphocytes and myeloid cells from the bloodstream into the CNS across leaky cerebrovascular venules (1). In contrast, progressive MS (PMS) involves an insidious, gradual decline in neurological function (most often in the form of worsening paraparesis, gait imbalance, and/or dementia), in the absence of focal blood-brain barrier (BBB) breakdown or influx of peripheral immune cells. Typical pathological features of PMS include slowly expanding white matter lesions, composed of a gliotic core surrounded by a rim of activated microglia, as well as widespread microglia activation in the macroscopically normal-appearing white matter (1, 2). Disease-modifying therapies that are approved for the treatment of MS mostly target lymphocytes in the peripheral immune system. While highly efficacious in RRMS, they have a modest therapeutic impact, at best, in PMS. There is an unmet need for new treatments that slow, or even block, the accumulation of disability in individuals with PMS. The identification of novel therapeutic targets pertinent to PMS will require a deeper understanding of the pathogenic effector cells, mediators, and pathways specific to the manifestation of progressive disease.

Biological aging is the strongest factor associated with MS clinical phenotype. RRMS typically presents in the third or fourth decade, while the mean age of onset of PMS is 45 years old (3). The age of onset of PMS is similar whether it occurs at clinical presentation (referred to as primary progressive MS) or follows an initial RR phase (secondary progressive MS). PMS is rare before age 40 and virtually nonexistent in the pediatric population (4). MS relapse rates tend to decline with disease duration and chronological age (5). Collectively, these data suggest that physiological changes associated with biological aging interact with the autoimmune response that drives CNS damage during MS, in a manner that transforms the clinical course and underlying neuropathology into a progressive pattern. Biological aging could affect the pathogenesis of MS via direct effects on immune cells or CNS-resident cells. Regarding the former, aging leads to immunosenescence, including a chronic, low-grade inflammatory state referred to as “inflamm-aging.” Regarding the latter, aging CNS tissues may be more vulnerable to inflammatory insults and less capable of initiating repair. The relative contributions of these aging-related phenomena to chronic active lesions, widespread microglial activation, and relentless accumulation of disability during PMS remain to be elucidated.

Experimental autoimmune encephalomyelitis (EAE), the most popular animal model of MS, generally presents with an acute monophasic or RR course (6). The hallmark pathological features of EAE, such as multifocal BBB breakdown and CNS influx of hematogenous leukocytes, are more reminiscent of RRMS than PMS. EAE is traditionally induced in young adult mice, between 8–12 weeks of age (7). There is a relative dearth of publications examining EAE in middle-aged or geriatric mice. A limited number of previous studies have shown that clinical EAE, induced by active immunization, is more severe and chronic in geriatric C57BL/6 or Biozzi ABH mice when compared with syngeneic young adult mice (8, 9). The cellular and molecular mechanisms underlying such age-related differences in disease severity are poorly understood. In the current study, we revisit the impact of biological aging on autoimmune neuroinflammation by comparing the clinical course, neuropathology, and inflammatory milieu in young adult versus middle-aged Th17 adoptive transfer recipients.

## Results

### Biological aging exacerbates the effector phase of EAE.

**Figure 1:**
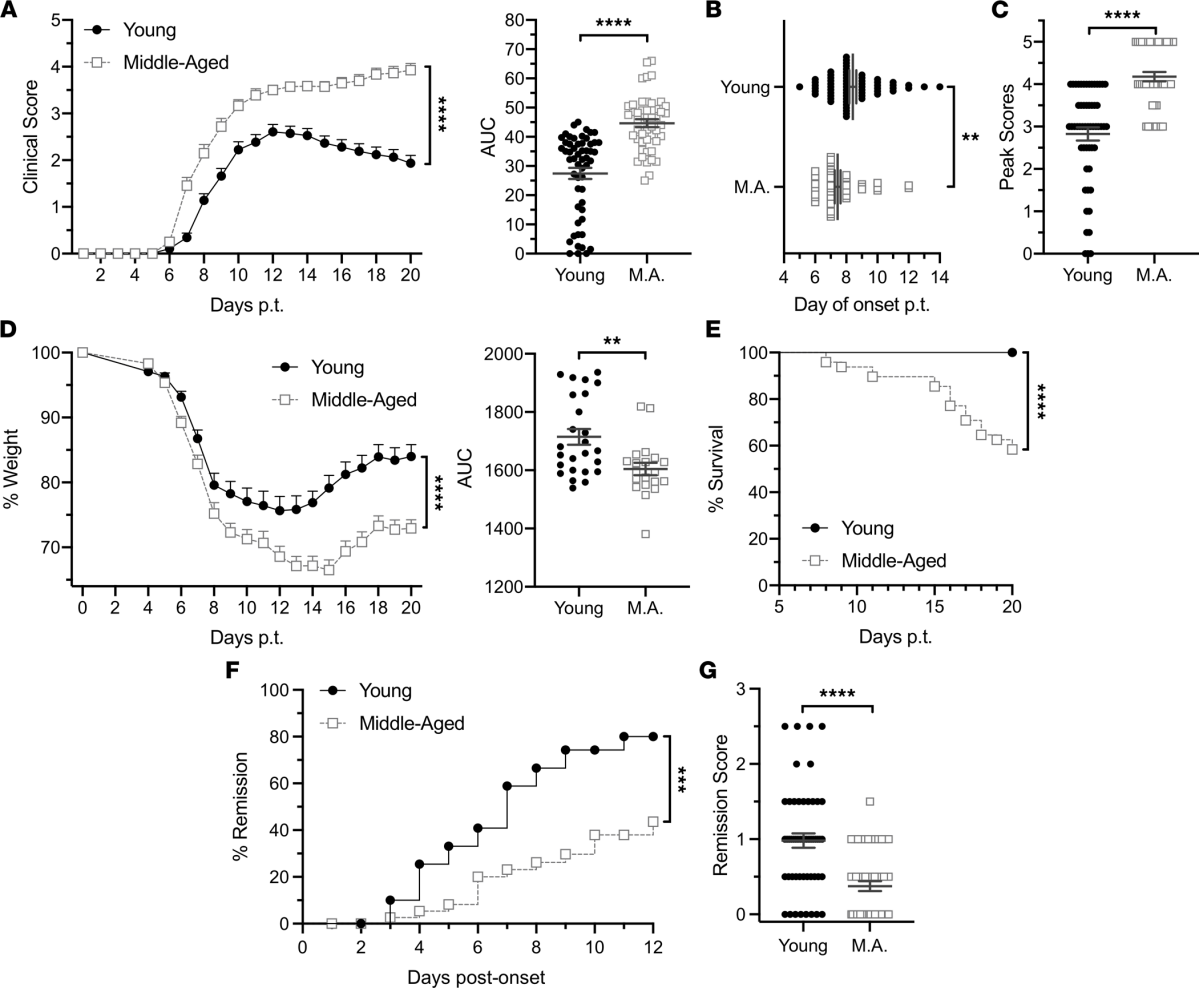
Middle-aged mice exhibit an exacerbated, nonremitting course of Th17-mediated EAE. EAE was induced by the adoptive transfer of myelin-reactive Th17 cells into young or middle-aged mice. (**A**–**C**) Young (*n* = 70) and middle-aged (*n* = 62) adoptive transfer recipients were scored daily for severity of neurological deficits while blinded to the identity of experimental groups. Data were pooled from 4 independent experiments. (**A**) Mean clinical scores of young and middle-aged hosts over time (left panel). The AUC was measured for individual mice in each group (right panel). (**B**) Day of disease onset posttransfer (p.t.) and (**C**) peak clinical disease scores of individual mice. (**D**) Young (*n* = 40) and middle-aged (*n* = 36) recipients were weighed daily. Data were pooled from 3 independent experiments. The percentage of baseline weight over time, averaged across young and middle-aged recipients (left panel). The AUC was measured for individual mice in each group (right panel). (**E**) Percentage survival over the disease course. (**F**) Percentage of mice undergoing remission on each day after clinical onset. (**G**) Remission score was calculated for each mouse by subtracting the clinical score at study termination (day 20 after cell transfer) from the clinical score at peak disease severity. Each symbol in **B**, **C**, and **G**, and the right panels of **A** and **D**, represents data generated from a single mouse. Statistical significance was determined using unpaired 2-tailed Student’s *t* test. Curves in the left panels of **A** and **D** were compared using a mixed effects model. Curves in **E** and **F** were compared using the log-rank test. Error bars indicate mean ± SEM. *P < 0.05, **P < 0.01, ***P < 0.001, ****P < 0.0001.

**Figure 2:**
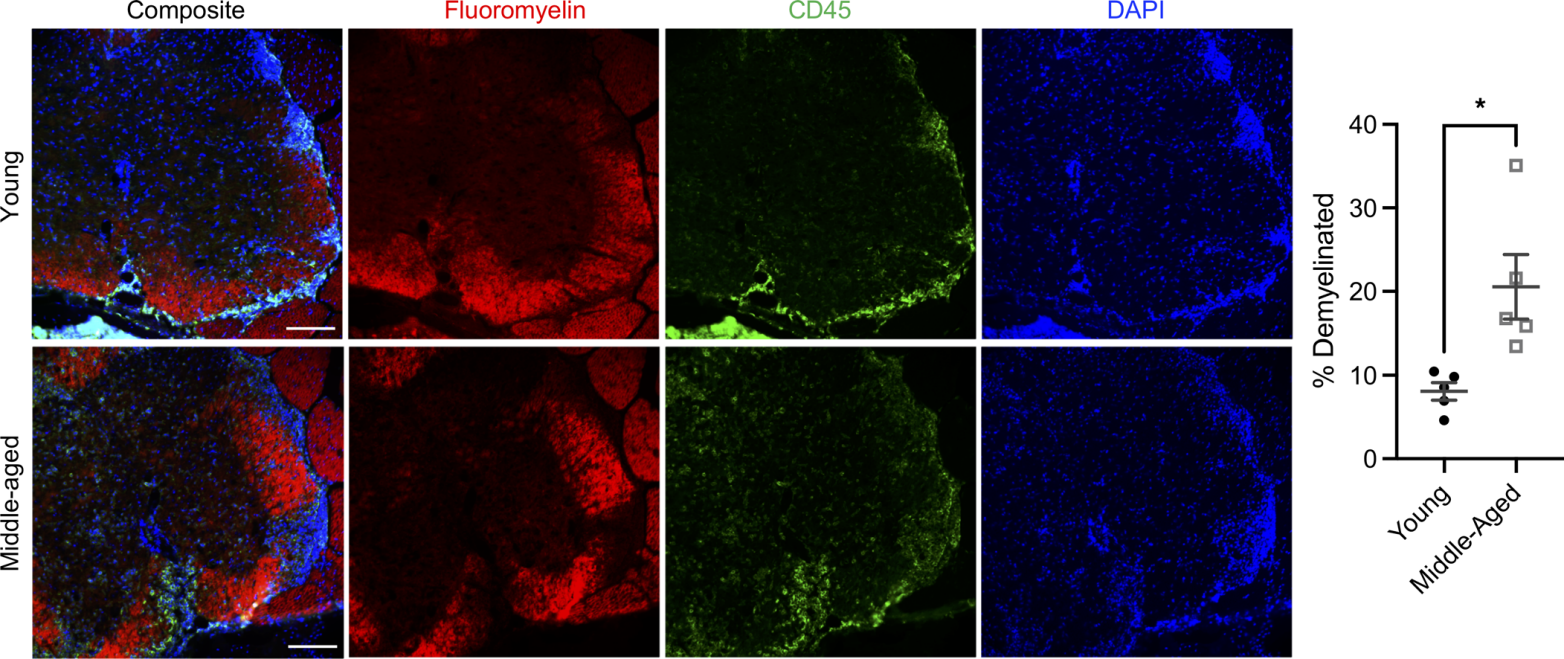
Spinal cord demyelination and inflammatory infiltration are exacerbated in middle-aged recipients. Representative spinal cord sections of young (upper panels) and middle-aged (lower panels) adoptive transfer recipients of encephalitogenic Th17 cells. Sections were stained for myelin (Fluoromyelin; shown in red), CD45 (green), and DAPI (blue). Demyelinated area is shown as a percentage of total white matter area. Data were pooled from 2 independent experiments with 5 mice per group. All images were acquired at 20× original magnification. Scale bars represent 100 microns. Statistical significance was determined using the unpaired 2-tailed Student’s *t* test. *P < 0.05.

**Figure 3:**
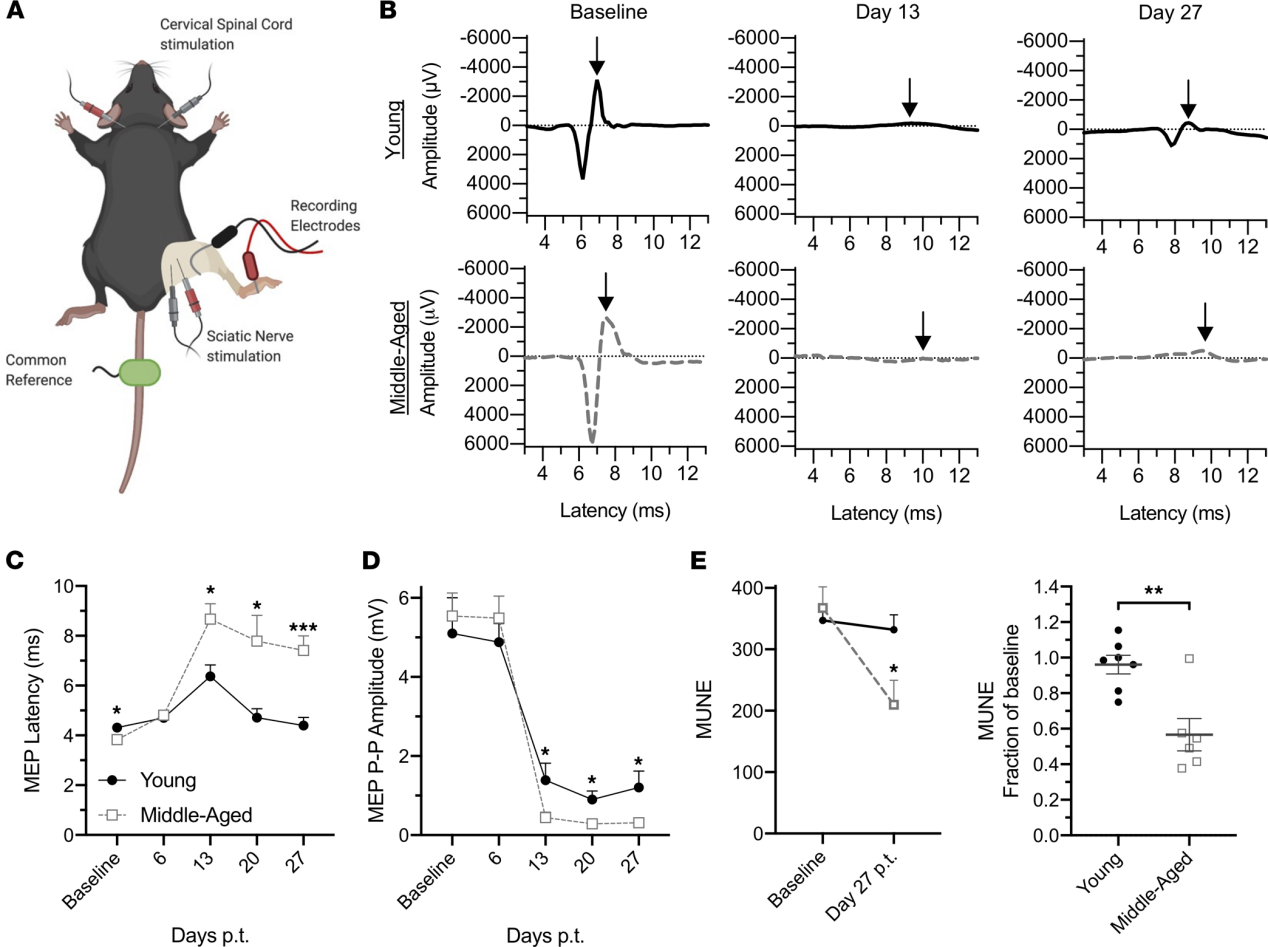
Electrophysiological studies indicate increased demyelination and neuronal/axonal loss in the spinal cords of middle-aged mice with EAE. (**A**–**E**) EAE was induced in young adult (*n* = 10) and middle-aged (*n* = 10) mice by the adoptive transfer of encephalitogenic MOG-specific CD4^+^ T cells. Mice were anesthetized for electrographic recordings. (**A**) Depiction of electrode placement. (**B**) Representative cervical motor-evoked potentials recorded from the right gastrocnemius following cervical spinal cord stimulation at baseline, day 13, and day 27 after cell transfer. Arrows indicate negative peaks of the cervical motor-evoked potentials. (**C** and **D**) Electrographic measurements were obtained at baseline and then weekly posttransfer (p.t.). Measurements of motor-evoked potential latency (**C**) and peak-to-peak (P-P) amplitude (**D**) were averaged across each group. (**E**) Motor unit number estimation (MUNE) was calculated at baseline and on day 27 posttransfer. Mean values for each group are shown (left panel). The ratio of MUNE at day 27 after cell transfer over baseline is shown for individual mice (right panel). Statistical significance determined by unpaired 2-tailed Student’s *t* test. *P < 0.05, **P < 0.01, ***P < 0.001. Error bars indicate mean ± SEM.

In order to differentiate between the effects of biological aging during the T cell–priming and effector stages of EAE, we used an adoptive transfer model in which IL-23–polarized, MOG_35-55_–reactive CD4^+^ T cells from donors of different ages are injected into naive syngeneic recipients of different ages. Traditionally, EAE is induced in young adult mice, between 8 and 12 weeks of age. For the middle-aged cohort, we used mice 40–44 weeks of age, based on their general developmental similarity to humans at the mean age of onset for PMS (10). We found no significant differences in the clinical course of EAE induced by encephalitogenic CD4^+^ T cells derived from MOG-immunized young adult versus middle-aged donors (Supplemental Figure 1; supplemental material available online with this article; https://doi.org/10.1172/jci.insight.158153DS1). In contrast, the clinical course of EAE was accelerated and exacerbated in middle-aged, compared with young adult, adoptive transfer recipients that had been injected with the same pool of encephalitogenic CD4^+^ T cells (Figure 1). Middle-aged Th17 cell recipients developed neurological deficits earlier, had higher peak scores, and had more cumulative neurological disability (Figure 1, A–C). Middle-aged recipients also exhibited greater weight loss, and a significant proportion succumbed to disease (58%), while all young recipients survived (Figure 1, D and E). Moreover, approximately 80% of young adult recipients underwent clinical remission, compared with approximately 40% of their middle-aged counterparts (Figure 1F), and they experienced a greater degree of recovery (Figure 1G). These age-related differences in the clinical course were reproducible across male and female cohorts (Supplemental Figure 1G). Immunohistochemical analysis of spinal cord sections revealed more extensive demyelination, associated with parenchymal infiltration by CD45^hi^ hematogenous leukocytes, in middle-aged adoptive transfer recipients (Figure 2). Electrophysiological studies were performed to assess the functional integrity of the spinal cord (Figure 3, A–E). Motor-evoked responses following cervical spinal cord stimulation showed delayed responses in all mice at peak and chronic stages of EAE compared with baseline, indicative of demyelination in the corticospinal tracts (Figure 3B). By week 2 posttransfer, the delay in nerve signal propagation was significantly longer in middle-aged recipients (Figure 3C), consistent with enhanced myelin damage, as had also been revealed by immunohistochemistry. In addition, from day 13 posttransfer onward, middle-aged mice exhibited a greater reduction in motor-evoked potential amplitudes compared with young adult mice, signifying increased axonal dysfunction and/or loss (Figure 3D). MUNE is calculated by dividing the maximum compound muscle action potential amplitude, obtained with sciatic nerve stimulation, by the average single motor unit potential. MUNE, which reflects the number of motor units innervating single muscles, was lower in the sciatic innervated hind limb muscles of middle-aged recipients on day 27 posttransfer (Figure 3E), consistent with a greater loss or dysfunction of motor neurons in the lumbar spinal cord.

### Encephalitogenic T cells and neutrophils are expanded, while B cells are retracted, in CNS infiltrates of middle-aged mice with EAE.

**Figure 4:**
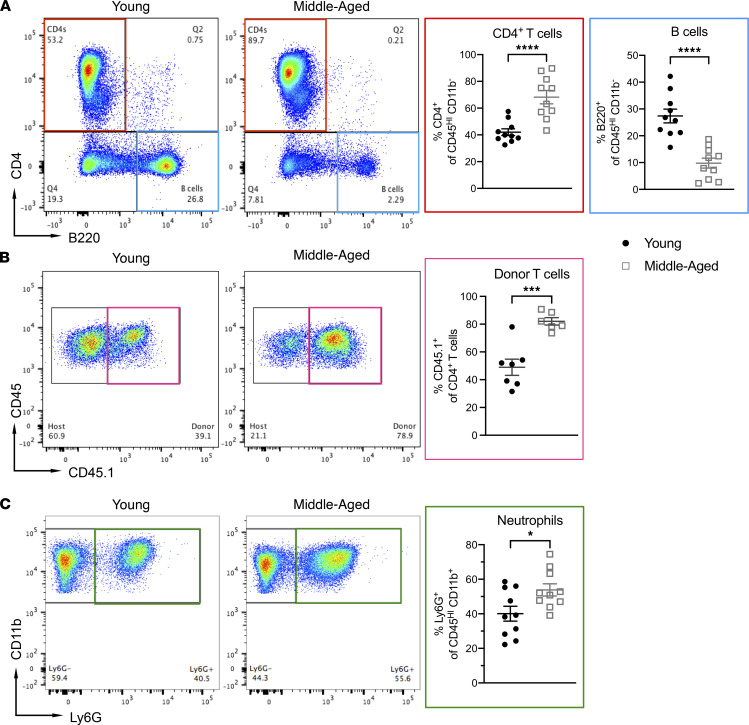
The composition of CNS-infiltrating immune cells differs between middle-aged and young adult mice with EAE. (**A**–**C**) Mononuclear cells were isolated from the spinal cords of young adult and middle-aged mice on day 10 posttransfer of encephalitogenic CD45.1^+^CD4^+^ T cells for flow cytometric analysis, gating on all CD45^+^ cells. Representative dot plots (left panels). The frequencies of CD4^+^ T cells and B cells (**A**), CD45.1^+^ donor CD4^+^ T cells (**B**), and neutrophils (**C**), among CNS-infiltrating CD45^+^ cells (right panels). Each symbol represents an individual mouse. Data were pooled from 3 independent experiments with a total of 7–10 mice per group. Statistical significance was determined using the unpaired 2-tailed Student’s *t* test. *P < 0.05, ***P < 0.001, ****P < 0.0001. Error bars indicate mean ± SEM.

**Figure 5:**
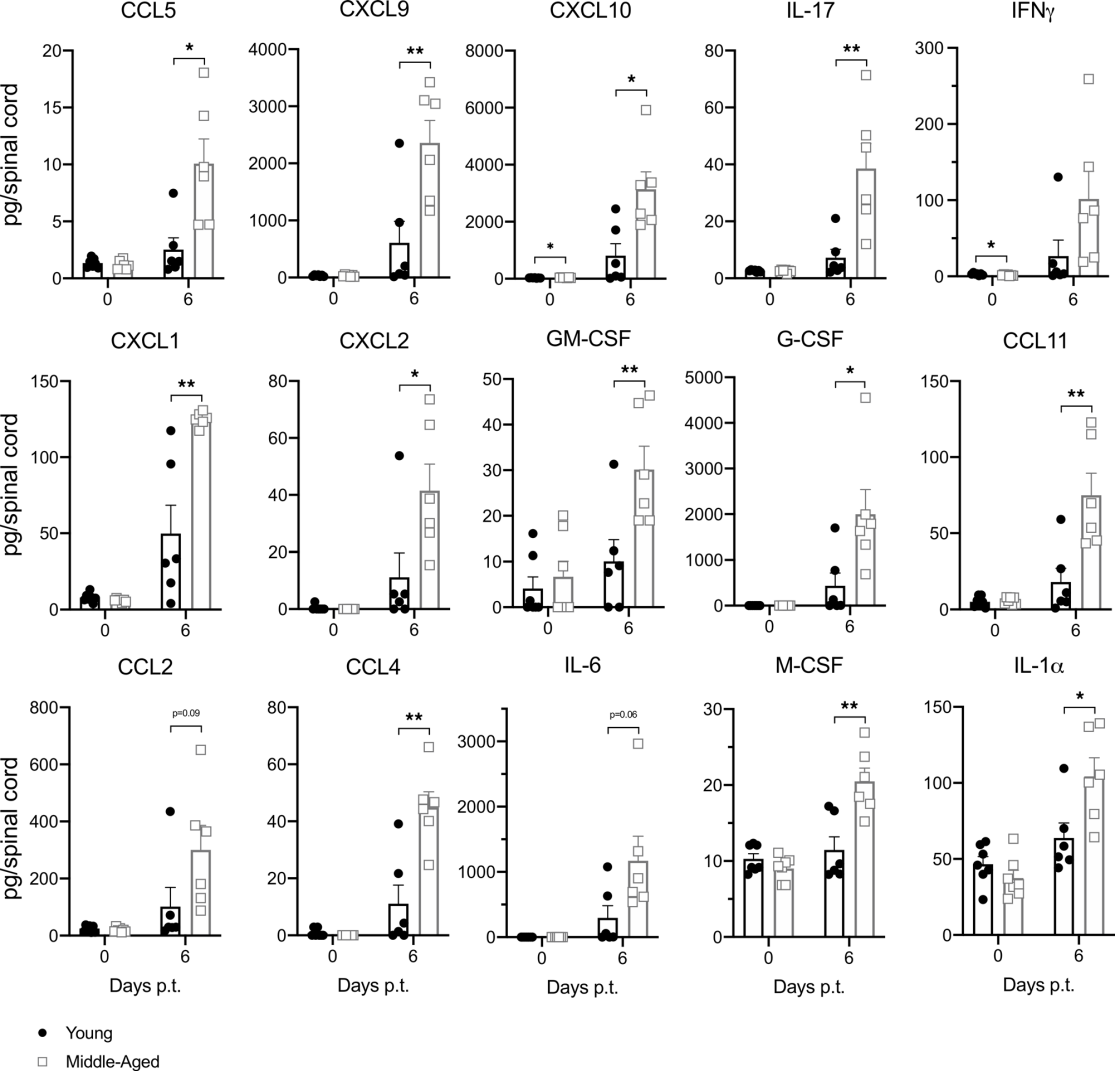
Proinflammatory proteins are elevated in the spinal cords of middle-aged mice with adoptive transfer EAE. Spinal cord homogenates were obtained from young (closed circles) and middle-aged (open squares) naive mice (left bars) or adoptive transfer recipients on day 6 posttransfer (right bars). A panel of proinflammatory factors were measured using the Luminex bead-based multiplex platform. Data were pooled from 2–4 independent experiments with a total of 7–13 mice/group. Each symbol represents a single mouse. Statistical significance was determined using the unpaired 2-tailed Student’s *t* test. *P < 0.05, **P < 0.01. Error bars indicate mean ± SEM.

The exacerbated clinical course of EAE in older mice could be secondary to a more virulent neuroimmune response, increased susceptibility of CNS-resident cells/myelin to immune-mediated damage, or a combination of these mechanisms. To investigate the former possibility, we compared the cellular composition of neuroinflammatory infiltrates between middle-aged and young adult mice on day 10 after T cell transfer (representative gating strategy shown in Supplemental Figure 2). There were significantly higher frequencies of CD4^+^ T cells and neutrophils, and a lower frequency of B cells, among CD45^+^ cells isolated from the spinal cords of the middle-aged adoptive transfer recipients (Figure 4, A–C). Frequencies of macrophages/monocytes or monocyte-derived dendritic cells did not differ between the groups (Supplemental Figure 3A). Similar patterns were observed with regard to the absolute numbers of immune cell subsets per spinal cord (Supplemental Figure 3B). For our experiments, we routinely obtain MOG_35-55_-primed CD4^+^ T cells from CD45.1^+^ congenic donors, in order to distinguish the transferred encephalitogenic T cells from CD45.2^+^ bystander host T cells. We found that CD45.1^+^CD4^+^ T cells were disproportionately expanded in the CNS of middle-aged recipients (Figure 4B). A broad panel of proinflammatory and chemotactic factors were upregulated in spinal cord lysates of all mice with EAE but were consistently elevated to higher levels in older mice. These factors include the chemokines CCL5, CXCL9, and CXCL10, which have been shown to target encephalitogenic T cells (11, 12), and CXCL1 and CXCL2, the major chemoattractants of mature neutrophils (13, 14) (Figure 5). Neutrophil-mobilizing factors GM-CSF and G-CSF were also expressed at higher levels in spinal cord lysates obtained from older mice.

### GM-CSF promotes the early, but not late, stage of exacerbated EAE in aged mice.

**Figure 6:**
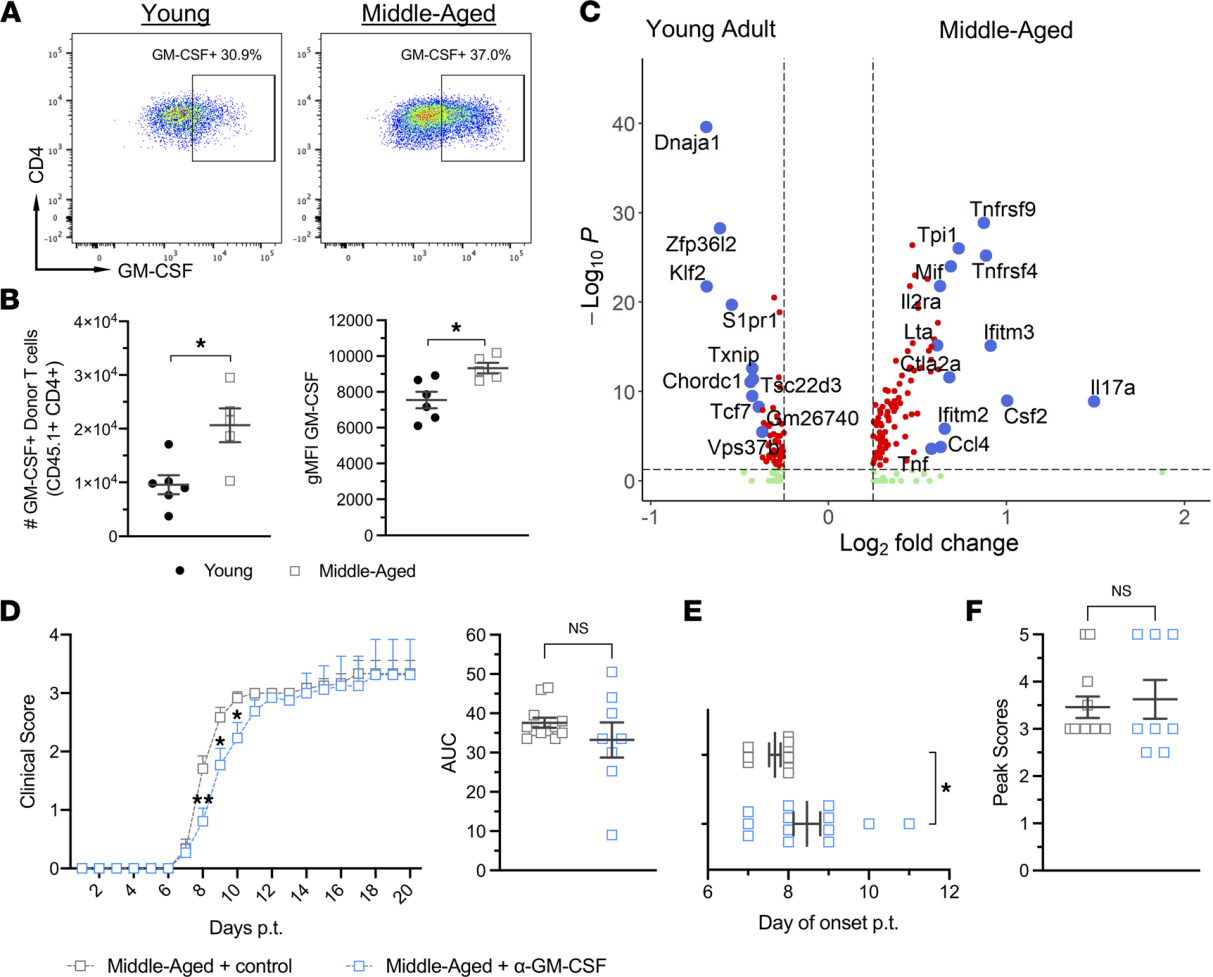
GM-CSF promotes exacerbated EAE in middle-aged mice early, but not late, in the clinical course. (**A** and **B**) CNS mononuclear cells were harvested from the spinal cords of young adult and middle-aged mice on day 10 after T cell transfer for analysis by flow cytometry. (**A**) Representative dot plots showing intracellular GM-CSF expression, gating on CD45.1^+^ donor CD4^+^ T cells. (**B**) Total number of GM-CSF^+^ donor T cells (left panel), and geometric mean fluorescence intensity (gMFI) of GM-CSF in donor T cells (right panel), isolated from the spinal cords of individual mice. Data were pooled from 2 independent experiments with 5–6 mice/group. (**C**) CD45^+^ mononuclear cells, FACS sorted from the spinal cords of young adult or middle-aged mice with EAE on day 6 after cell transfer, were analyzed by single-cell RNA sequencing (scRNA-Seq). The volcano plot shows gene expression changes, comparing young versus middle aged cells, within the CD4^+^ T cell cluster. Red dots represent genes that are below an adjusted *P* value of 0.05 and greater than an absolute log_2_-fold change of 0.25. Green dots show genes that meet the fold change threshold but not the *P* value threshold. Blue dots show manually selected genes to display. (**D**–**F**) Middle-aged mice were injected i.p. with anti–GM-CSF neutralizing antibody or control antibody every other day from the time of encephalitogenic T cell transfer onward (*n* = 14 mice/group; pooled from 2 independent experiments). Mice were scored for severity of neurological deficits on a daily basis while blinded to the identity of the experimental groups. (**D**) Mean clinical scores of mice in each group over time (left panel). AUC of individual mice (right panel). (**E**) Day of clinical disease onset posttransfer and (**F**) peak clinical disease scores of individual mice. Statistical significance was determined using the unpaired 2-tailed Student’s *t* test. *P < 0.05 or as indicated. Error bars indicate mean ± SEM.

We have previously shown that GM-CSF receptor–deficient (GM-CSFR–deficient) mice are relatively resistant to adoptively transferred EAE (15). They experience a milder course than wild-type counterparts, with an increased rate of remission. In addition, GM-CSFR deficiency is associated with a lower percentage of CD4^+^ donor T cells and neutrophils, and a higher percentage of B cells, in CNS infiltrates, which is the mirror image of the pattern that we observed in middle-aged, wild-type mice with EAE. Collectively, these observations suggest that the exacerbated form of EAE that occurs in middle-aged, wild-type mice is GM-CSF driven. Indeed, the absolute number of GM-CSF–expressing donor CD4^+^ T cells in CNS infiltrates, the level of intracellular GM-CSF in CNS donor T cells, and the level of GM-CSF protein in CNS lysates, were higher in the older versus younger mice with EAE (Figure 5 and Figure 6, A and B). Furthermore, CD4^+^ T cells isolated from the CNS of middle-aged mice on day 6 posttransfer expressed elevated levels of transcripts encoding GM-CSF, as well as IL-17 and other proinflammatory factors, compared with their younger counterparts, based on single-cell RNA-sequencing analyses (Figure 6C). Treatment of middle-aged hosts with a neutralizing antibody against GM-CSF, beginning from the day of transfer onward, delayed clinical onset and ameliorated the early clinical course (Figure 6, D and E). However, mean peak scores, cumulative chronic disability, and mortality rates were similar between the anti–GM-CSF and control antibody treatment groups (Figure 6, D and F, and data not shown). Postponing the initiation of anti–GM-CSF treatment to the time of peak disease had no therapeutic impact (data not shown). Based on these data, we concluded that GM-CSF promotes aggressive EAE in older recipients in the early phase of disease but becomes dispensable as the disease progresses toward a more chronic phase.

### Radio-resistant, nonhematopoietic cells shape the neuroinflammatory infiltrate and drive exacerbated EAE in middle-aged mice.

**Figure 7:**
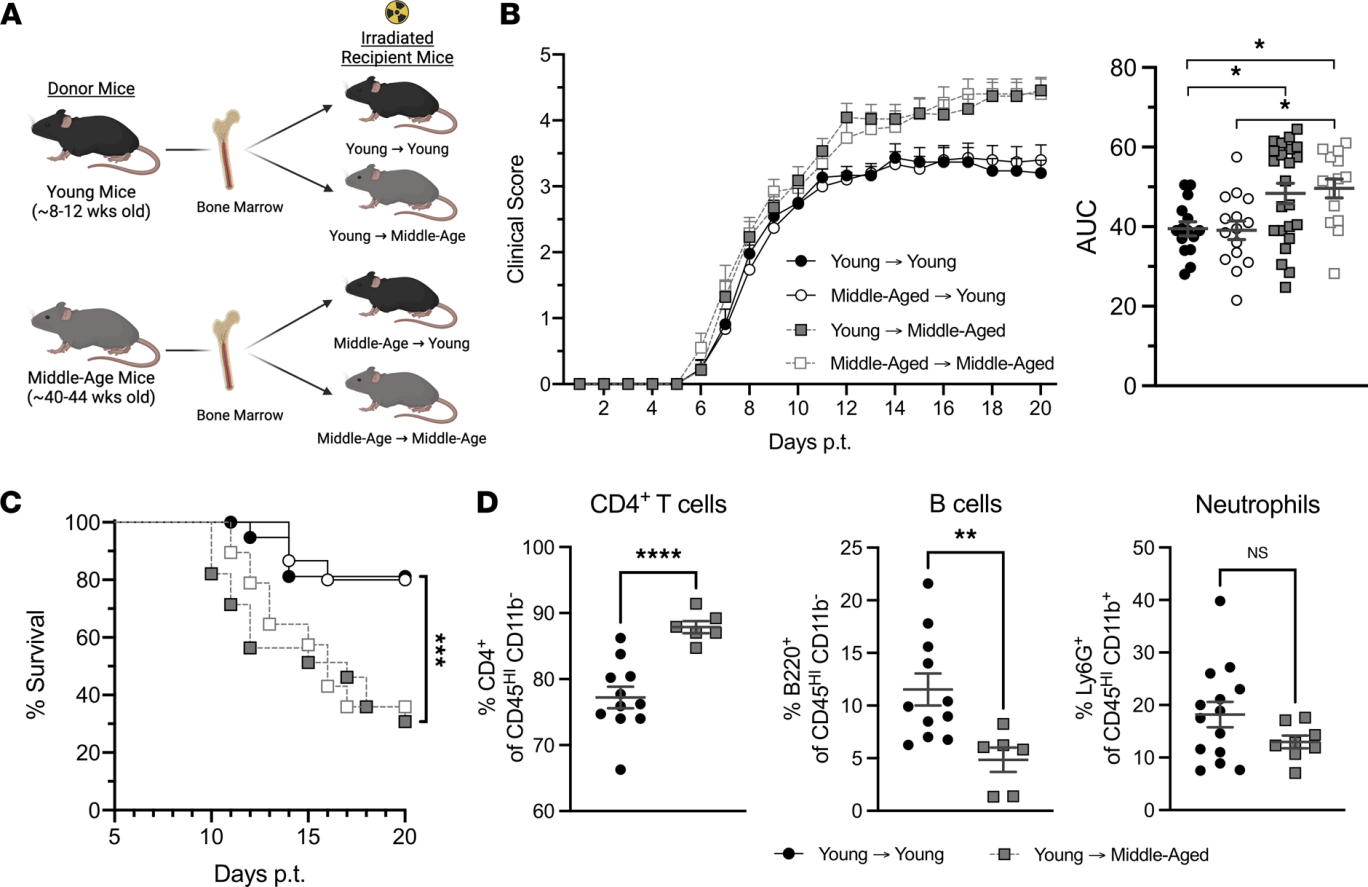
Radio-resistant, nonhematopoietic cells drive exacerbated EAE in middle-aged mice. (**A**–**D**) Reciprocal bone marrow chimeric mice were constructed with young adult or middle-aged donors and hosts. Following reconstitution, all chimeric mice were injected i.p. with 3 × 10^6^ MOG-primed CD4^+^ Th17 cells. Mice were scored for severity of neurological deficits on a daily basis while blinded to the identity of the experimental groups. (**A**) Schematic depicting the construction of reciprocal bone marrow chimeras. (**B**) Mean clinical scores over time (left panel) and AUC of individual mice (right panel). (**C**) Percentage of surviving mice in each group over time. (**D**) Frequencies of CD4^+^ T cells, B cells, and neutrophils among CD45^+^ spinal cord mononuclear cells harvested from individual mice on day 10 posttransfer. Data in **B** and **C** were pooled from 3 independent experiments with a total of 15–28 mice per group. Each symbol in **B**, right panel, and in **D** represents an individual mouse. Data in **D** are pooled from 3 independent experiments with a total of 6–14 mice/group. Statistical significance was determined using a 1-way ANOVA and Dunnett’s T3 multiple-comparison test in **B** and unpaired 2-tailed Student’s *t* test in **D**. Curves in **C** were compared using the log-rank test. *P < 0.05, **P < 0.01, ***P < 0.001, ****P < 0.0001. Error bars indicate mean ± SEM.

Next, we sought to determine whether the increased susceptibility of older mice to EAE is secondary to the biological aging of radio-sensitive, hematopoietic cells (that are recruited from the circulation to the CNS) and/or radio-resistant, nonhematopoietic cells (including CNS-resident cells). To that end, we constructed reciprocal bone marrow (BM) chimeric mice with young adult or middle-aged BM cell donors and/or irradiated hosts (Figure 7A). Middle-aged → middle-aged and young adult → young adult chimeras served as controls. EAE was induced in all immune-reconstituted chimeric mice via the adoptive transfer of the same pool of MOG_35-55_ encephalitogenic CD4^+^ T cells. Irrespective of the age of the BM cell donors, middle-aged BM cell hosts experienced a severe clinical course of EAE, with high mortality rates, while young adult BM cell hosts experienced a milder course with relatively low mortality rates (Figure 7, B and C). Furthermore, the cellular composition of neuroinflammatory infiltrates in chimeras correlated with the age of the BM cell host, but not the donor, mouse. Middle-aged BM cell hosts reconstituted with young BM cells contained higher frequencies and absolute numbers of CD4^+^ T cells, and lower frequencies and absolute numbers of B cells, in EAE lesions compared with chimeras that were constructed using young adult hosts, mimicking the results we obtained with nonchimeric mice (Figure 7D and Supplemental Figure 3C). Hence, the age of radio-resistant, nonhematopoietic cells is a dominant factor in determining the phenotype and severity of EAE.

### Aged microglia exhibit distinct transcriptomes and phenotypes during homeostasis, as well as EAE.

**Figure 8:**
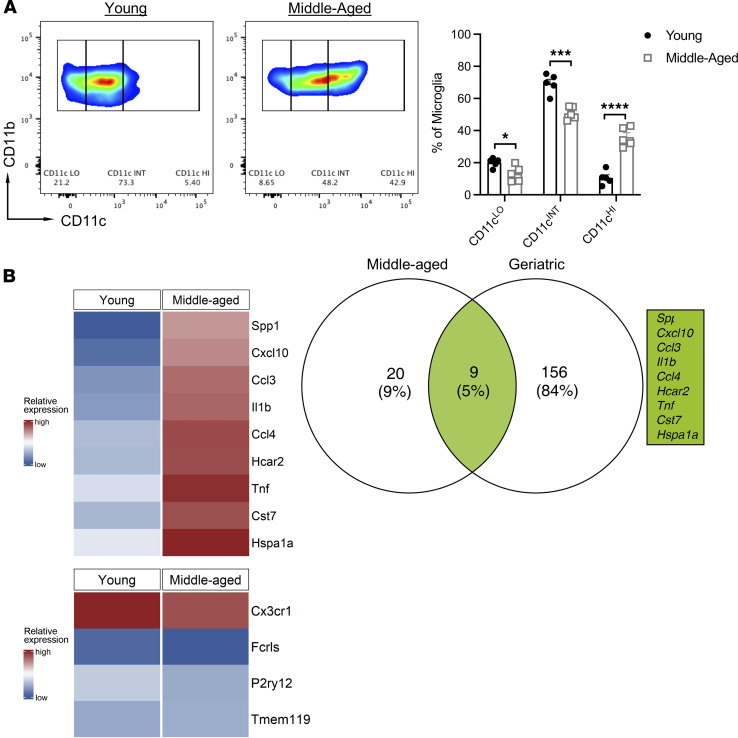
Microglia in middle-aged mice upregulate genes indicative of a reactive, age-associated phenotype. Mononuclear cells were harvested from the spinal cords of naive young adult and middle-aged mice. (**A**) Representative dot plots (left and middle panel), and frequencies of CD11c^lo^, CD11c^int^, and CD11c^hi^ cells among CD45^int^CD11b^+^ microglia (right panel), are shown. Each symbol in the right panel represents an individual mouse. Data were pooled from 2 independent experiments with *n* = 5 mice/group. Statistical significance was determined using the unpaired 2-tailed Student’s *t* test. *P <0.05, ***P < 0.001, ****P < 0.0001. Error bars indicate mean ± SEM. (**B**) CD45^int^CD11b^+^ cells were FACS sorted, and RNA was extracted for bulk sequencing. Venn diagram depicts the number of overlapping genes and overall percentage of genes upregulated in naive microglia isolated from middle-aged mice compared with geriatric mice (age P540) (left). Heatmaps illustrating the relative expression of overlapping genes (right) and homeostatic genes (bottom).

Our finding that radio-resistant cells influence the makeup of neuroinflammatory infiltrates directed our attention toward the role of microglia. In addition to their potential to serve as antigen-presenting cells, microglia produce chemotactic and proinflammatory molecules that could orchestrate the recruitment, positioning, and polarization of leukocyte subsets in EAE lesions. A growing body of data indicates that microglia become spontaneously activated and acquire proinflammatory signatures with age (16, 17). In support of these published studies, we found that a significant percentage of CD45-intermediate (CD45^int^) CD11b^+^ microglia in naive middle-aged, but not young adult, mice exhibited enhanced expression of the cell surface marker CD11c (Figure 8A and Supplemental Figure 2). Bulk RNA-sequencing studies showed that microglia isolated from unmanipulated, middle-aged mice expressed elevated levels of proinflammatory genes and lower levels of homeostatic genes (Figure 8B), compared with microglia from their younger counterparts. Furthermore, a number of genes that were disproportionately upregulated in the middle-aged microglia (such as S*pp1*, C*xcl10*, *Ccl3*, *Il1β*, *Ccl4*, and *Tnf*) overlapped with previously published transcriptomes of aging microglial subsets enriched in geriatric mice (Figure 8B) (16).

**Figure 9:**
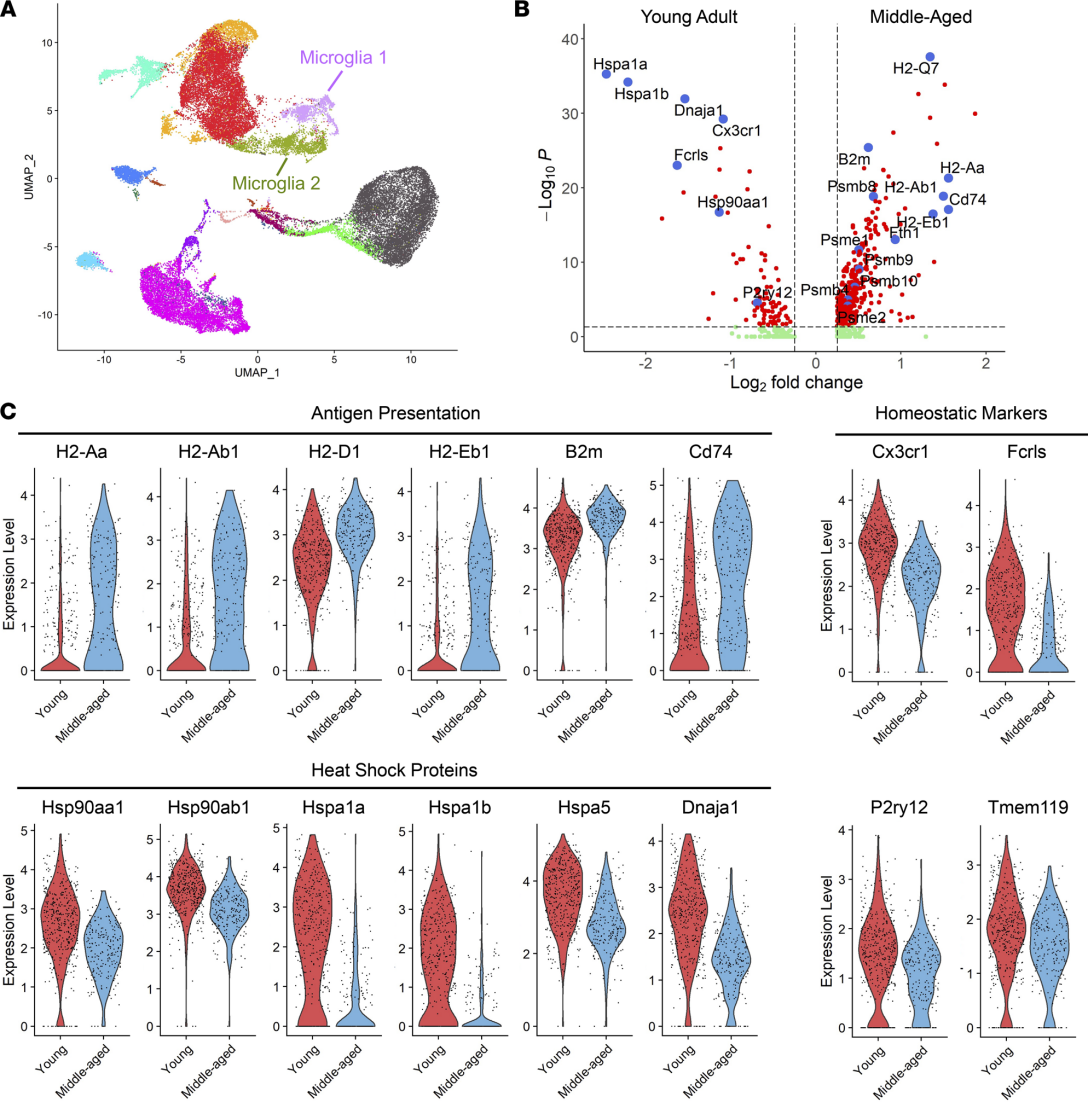
Microglia from middle-aged mice with EAE express distinct transcriptomic signatures. CD45^+^ mononuclear cells were FACS sorted from young adult or middle-aged mice with EAE on day 6 after cell transfer and subjected to scRNA-Seq. (**A**) Uniform manifold approximation and projection (UMAP) plot showing clustering by cell type, labeled on the basis of known lineage markers. (**B**) Gene expression changes in cluster 1 microglia isolated from young adult versus middle-aged mice, shown as a volcano plot. Red dots represent genes that are below an adjusted *P* value of 0.05 and greater than an absolute log_2_-fold change of 0.25. Green dots show genes that meet the fold change threshold but not the *P* value threshold. Blue dots show manually selected genes to display. (**C**) Selected differentially expressed genes in cluster 1 microglia from young adult (red) and middle-aged (blue) adoptive transfer recipients, shown as violin plots. Numbers on the *y* axis correspond to the *z* score for each gene.

**Figure 10:**
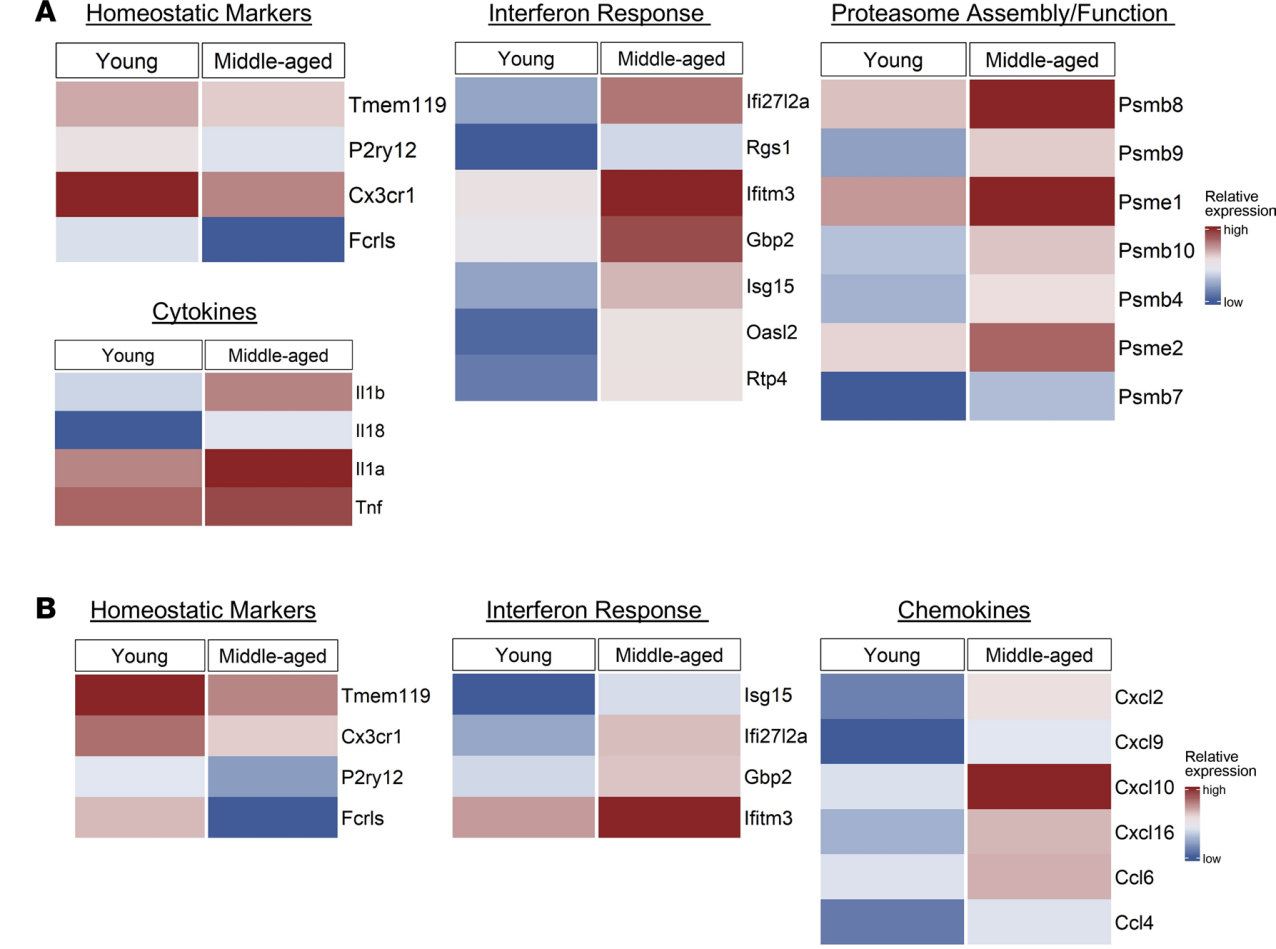
Microglia from middle-aged mice with EAE downregulate homeostatic genes and upregulate proinflammatory and IFN response genes. scRNA-Seq was performed on CD45^+^ CNS mononuclear cells isolated from young adult and middle-aged adoptive transfer recipients as described in the legend to Figure 9. The heatmaps show *z* score–transformed normalized expression values of selected genes in cluster 1 microglia (**A**) or cluster 2 microglia (**B**), comparing young adult and middle-aged recipient mice.

Next, we performed scRNA-Seq of CNS CD45^+^ mononuclear cells isolated from young adult and middle-aged mice during peak EAE. The raw scRNA-Seq expression matrix contains 38,057 cells (including 19,899 cells from middle-aged mice and 18,158 cells from young adult) and 19,449 genes. The microglial cells fell into 2 clusters (Figure 9A). Cluster 1 microglia from middle-aged mice were enriched in transcripts associated with antigen processing and presentation (including *H2-Aa*, *H2-Ab1*, *H2-D1*, *H-2-Eb1*, *H2K1*, *H-2Q7*, *B2m*, *Cd74*), and proteasome assembly and function (*Psmb4*, *Psmb8*, *Psmb9*, *Psmb10*, *Psme1*, *Psme2*), as well as transcripts that encode proinflammatory molecules (*Tnf*, *Il1a*, *Il1b*, *Il18*) (Figure 9, B and C, and Figure 10A), when compared with their counterparts from young adoptive transfer recipients. In contrast, cluster 1 microglia from middle-aged mice were relatively deficient in transcripts associated with homeostasis (*Tmem119*, *Fcrls*, *Cx3cr1*, *P2ry12*) and heat shock protein responses (*Dnaja1*, *Dnajb1*, *Hspa1a*, *Hspa1b*, *Hspa5*, *Hsp90aa1*, *Hsp90ab1*) (Figure 9, B and C, and Figure 10A). Cluster 2 microglia from middle-aged mice expressed relatively high levels of genes encoding chemotactic factors (including *Ccl4*, *Cxcl2*, *Cxcl9*, and *Cxcl10*) and relatively low levels of genes associated with homeostasis (including *Fcrls*, *Tmem119*, *P2ry12*, and *Cx3cr1*) (Figure 10B). Middle-aged microglia in both clusters expressed relatively high levels of interferon responses genes (including *Gbp2*, *Ifitm3*, *Ifi27l2a*, and *Isg15*) (Figure 10, A and B), as well as genes that are expressed at high levels in microglia located at the rims of slowly expanding lesions in people with PMS (*C1qa*, *C1qb*, *C1qc*, *Cstb*, *Fth1*, *Ftl1*, and a panel of ribosomal proteins) (Supplemental Figure 4).

## Discussion

PMS typically presents during middle age, whether or not it is preceded by an RR course. It rarely, if ever, occurs in the pediatric MS population (4). PMS has distinctive pathological features that are not characteristic of RRMS in younger individuals, including widespread microglial activation (1). These observations indicate that biological aging has a profound influence on the evolution and manifestation of autoimmune neuroinflammation, but the underlying mechanisms are poorly understood. Here, we show that EAE, induced by the transfer of a common pool of IL-23–polarized, MOG-reactive CD4^+^ T cells, was more severe, and less likely to remit, in middle-aged compared with young adult recipients. We used an adoptive transfer model in order to differentiate between age-dependent environmental factors in the recipient mouse, as opposed to intrinsic properties of encephalitogenic T cells, that might contribute to the differences in EAE phenotype between the cohorts. The heightened, refractory disability older adoptive transfer recipients exhibited may reflect an increased vulnerability of aged oligodendrocytes, myelin, and/or axons to immune-mediated damage. However, our data indicate that aging has an impact on neuroinflammation itself, since the cellular composition of CD45^+^ cells in the CNS at peak EAE differed markedly between young adult and middle-aged recipients. Donor T cells and neutrophils were consistently more abundant, while B cells were relatively sparse, in the CNS of older mice. Our experiments with reciprocal BM chimeric mice indicated that radio-resistant, nonhematopoietic cells played a dominant role in shaping age-dependent features of the neuroinflammatory response, as well as the clinical course, during EAE. Among radio-resistant host cells, glia are strong candidates for regulators of CNS autoimmunity.

We found that murine microglia spontaneously upregulated CD11c as they aged. This is consistent with previous reports that aging microglia exhibit morphological, phenotypic, and transcriptomic changes indicative of a predisposition toward an activated or proinflammatory state (18–20). A number of the proinflammatory genes that we identified as upregulated in naive, middle-aged, compared with young adult, microglia have previously been reported to be upregulated in microglia from geriatric mice (14). Our working hypothesis is that the increased susceptibility of middle-aged mice to encephalitogenic T cell accumulation, white matter damage, and exacerbated clinical EAE, is driven, at least in part, by aging microglia. Indeed, enhanced microglial reactivity in the aging monkey brain correlates with an increase in perivascular T cell infiltrates in the white matter parenchyma, as well as cognitive impairment (21). Interestingly, genes we found to be highly expressed by microglia from middle-aged adoptive transfer recipients were also expressed at high levels by microglia located in the rims of chronic active lesions during PMS, including genes involved in expression of the MHC class II protein complex, ferritin complex, and complement cascade (Supplemental Figure 4).

The mechanisms underlying age-related changes in microglia are likely multifold. Neuronal maintenance of microglial homeostasis via CX3CL1/CX3CR1 and CD200/CD200R interactions wanes with aging (22–24). In addition, microglia can be stimulated by CNS-penetrant, proinflammatory factors, such as IL-6, CCL11, and IL-1β, that are systemically released by peripheral immune cells in the context of inflamm-aging, and/or by microbiome-derived molecules, such as LPS, that rise in the bloodstream consequent to age-related increases in gastrointestinal permeability (19, 20, 25–27). Microbial metabolites, which are altered during dysbiosis, also modulate microglial function in an age-dependent fashion. In young adults, gut microbiome-derived tryptophan metabolites and short chain fatty acids (SCFAs) cross the BBB and constitutively suppress microglia, thereby reducing the risk of neuroinflammation (28, 29). Dietary supplementation with the SCFA propionic acid is associated with less inflammatory activity, disability progression, and brain atrophy in MS (30). However, production of SCFAs and tryptophan metabolites by gut bacteria declines with aging (31), thereby removing another check on microglial activation.

scRNA-Seq of CD45^+^ leukocytes, harvested from the CNS at peak disease, revealed 2 clusters of EAE-associated microglia, both of which exhibited dynamic, age-dependent transcriptomic signatures (Figure 9). In comparison with their counterparts harvested from young adult adoptive transfer recipients, cluster 1 microglia from middle-aged recipients were highly enriched in transcripts that encode molecules engaged in the presentation of antigen to T cells. This finding led us to hypothesize that more efficient antigen presentation by cluster 1 microglia might boost the proliferation of donor T cells, resulting in higher T cell frequencies within the CNS infiltrates of middle-aged mice. However, contrary to that hypothesis, we found comparable frequencies of Ki67^+^ donor T cells among CD45^+^ CNS mononuclear cells isolated from young adult or middle-aged mice at peak EAE (data not shown). Furthermore, CD45^int^CD11b^+^ microglia, purified from young adult or middle-aged mice at peak EAE, stimulated the proliferation of 2D2 cells (T cell receptor–transgenic CD4^+^ cells specific for MOG peptide) to a similar degree (data not shown). To further investigate this issue, in future experiments we will compare the proliferation of donor T cells, and the antigen-presenting/T cell–polarizing capacity of sharply defined microglial subsets, in young adult versus middle-aged hosts at multiple time points. Interestingly, some of the transcripts upregulated in cluster 1 microglia from middle-aged mice are involved in the assembly and function of proteasomes and processing of MHC class I–restricted peptides. Although our model of EAE is CD4^+^ T cell driven, CD8^+^ T cells are more prevalent than CD4^+^ T cells in human MS lesions, including in the chronic active lesions that are typical of PMS (32–34). The emergence of microglia particularly well equipped to activate CD8^+^ T cells might be relevant to the pathogenesis of PMS.

In middle-aged hosts, cluster 2 microglia are enriched in transcripts that encode a range of chemokines including CXCL2 and CXCL10. We and others previously showed that neutrophil migration to the CNS during EAE is dependent on CXCR2-binding chemokines (such as CXCL1 and CXCL2) (13, 14, 35, 36), while CD4^+^ T cell migration is dependent on CXCR3-binding chemokines (such as CXCL9, CXCL10, and CXCL11) (11). Elevated production of CXCL2 and CXCL10 by aged cluster 2 microglia might trigger preferential accumulation of donor T cells and neutrophils locally and thereby underlie the relative enrichment of those subpopulations in the CNS of middle-aged hosts during EAE. The translational significance of this finding is underscored by the observations that patients with PMS have a relatively high percentage of circulating CXCR3^+^ lymphocytes (37), and CXCR3^+^ lymphocytes preferentially accumulate in MS lesions (38). Furthermore, CXCL9 and CXCL10 are upregulated in PMS brains (33), and expression of CXCR3 on circulating CD8^+^ T cells correlates with MS lesion volume (39). There is also evidence that neutrophil-related factors and chemokines are dysregulated in PMS and correlate with clinical, as well as radiological, measures of CNS tissue damage (27).

In addition to sculpting the neuroinflammatory response via antigen presentation and chemokine production, aging microglia could modulate EAE by releasing soluble factors that directly damage oligodendrocytes and/or axons, such as reactive oxygen and nitrogen species, enzymes, and TNF family members (40, 41), and/or that drive the polarization of neurotoxic astrocytes, such as IL-1α and C1q (29, 42). Conversely, we found that aging microglia in cluster 1 downregulated expression of transcripts encoding heat shock protein 90, which has been implicated in neuroprotection, and heat shock protein 70, which has been associated with the suppression of neurotoxic astrocytes (43, 44). Increased susceptibility of oligodendrocytes and neurons/axons to immune-mediated insults, combined with impairment of microglial phagocytosis, might escalate CNS deposition of damage-associated molecular patterns (DAMPs) and cellular debris in middle-aged hosts. DAMPs and cellular debris incite the activation of microglia and infiltrating leukocytes, which could fuel a self-amplifying cycle of tissue destruction. Although the adoptive transfer model of EAE in middle-aged mice lacks a number of salient pathological features of PMS (i.e., slowly expanding, chronic active lesions and cortical lesions), it may be useful for investigating how biological aging alters inflammatory demyelinating disease in other ways that simulate PMS (i.e., diffuse microglial activation and a nonremitting clinical course) and for testing the efficacy of senolytic drugs, microglial suppressors, or other novel therapeutic interventions potentially beneficial in the PMS subpopulation.

## Methods

### Mice

CD45.1 congenic and wild-type C57BL/6 mice were obtained from Charles River Laboratories or the NIH/National Cancer Institute. Mice were housed in micro-isolator cages under specific pathogen–free conditions at The Ohio State University. Both male and female mice were used in experiments.

### Induction and assessment of EAE

Mice at 8 to 12 weeks of age were immunized subcutaneously with an emulsion consisting of 100 μg of MOG_35-55_ peptide (MEVGWYRSP-FSRVVHLYRNGK; Biosynthesis) emulsified in CFA (BD Difco), at 4 sites over the flanks. Inguinal, axial, and brachial lymph nodes and spleens were harvested from donor mice 10–14 days postimmunization and passed through a 70 μm strainer (Thermo Fisher Scientific) to obtain a single-cell suspension. The cells were cultured for 96 hours with MOG_35-55_ peptide (50 μg/mL) and Th17-polarizing factors as follows: recombinant murine (rm) IL-23 (8 ng/mL; R&D Systems), rmIL-1α (10 ng/mL; Peprotech), and anti–IFN-γ (10 μg/mL; clone XMG1.2, BioXCell). After 96 hours, the cells were harvested, washed, and resuspended in fresh media. CD4^+^ T cells were purified via positive-selection, magnetic-activated cell sorting using L3T4 magnetic microbeads (Miltenyi Biotec) per the manufacturer’s protocol. CD4^+^ T cells (90%–98% purity) were transferred via i.p. injection to naive C57BL/6 recipients. Recipient mice were weighed and observed daily for clinical disability and rated using a 5-point scale as described previously (35): briefly, 0.5, partial tail paralysis; 1, full tail paralysis; 1.5, hind limb weakness demonstrated by ability to correct from a prone position in <1 second; 2, hind limb weakness demonstrated by ability to correct from a prone position in >1 second; 2.5, hind limb weakness demonstrated by severe gait abnormality; 3, partial hind limb paralysis demonstrated by the inability to elevate hindquarters; 3.5 complete paralysis in 1 hind limb; 4, complete hind limb paralysis; 4.5, moribund; and 5, dead. Remission was defined as a reduction in clinical score from peak EAE by at least 0.5 points for 2 consecutive days or more. Remission score was calculated for each mouse by subtracting the clinical score at the time of study termination (day 20) from the score at peak disease severity.

### CNS mononuclear cell and homogenate collection

Mice were anesthetized with isoflurane and perfused with 1× PBS. The spinal cord was flushed through the spinal canal with PBS. Individual cords were homogenized with an 18-gauge needle in a protease inhibitor solution created using protease inhibitor cocktail tablets (Roche) per the manufacturer’s protocol (to a total volume 1.0 mL), then centrifuged at 800*g* for 5 minutes at 4°C, and the supernatant was collected and stored at –80°C. Tissue pellet was resuspended in a solution of HBSS with 1 mg/mL collagenase A (Roche) and 1 mg/mL DNase I (MilliporeSigma) and incubated at 37°C for 20 minutes. Mononuclear cells were separated from myelin via centrifugation at 800*g* and 4°C for 5 minutes in a 27% Percoll solution (GE Healthcare).

### Fluorescence immunohistochemistry

Spinal cords were harvested from mice perfused with 1× PBS and 4% paraformaldehyde (PFA). Tissue were postfixed with 4% PFA for 24 hours, washed with 1× PBS, decalcified with 0.5 M EDTA for 4–6 days, and cryopreserved with 30% sucrose solution at 4°C. Tissue was embedded in OCT (Scigen Scientific) for cryosectioning and stored at –80°C. Sections were blocked with 5% normal goat serum (MilliporeSigma), washed with 0.1% Triton X-100 (Thermo Fisher Scientific) in 1× PBS (PBS-T), and stained with rat anti–mouse CD45 (clone IBL-5/25; MilliporeSigma) for 48 hours at 4°C. Following washing with PBS-T, sections were stained with Alexa Fluor 647 goat anti–rat IgG (catalog A-21247, Invitrogen) for 2 hours. Sections were then stained with Fluoromyelin Red Fluorescent Myelin Stain (Invitrogen) per the manufacturer’s protocol. ProLong Gold antifade reagent with DAPI (Invitrogen) was applied immediately prior to applying the coverslip. Images were acquired using an Olympus IX83 inverted fluorescence microscope with cellSens Dimension software (Olympus). All microscopy settings, including laser power, gain, contrast, offset, gamma value, and exposure times, were consistent across channels throughout image acquisition. Total white matter area and areas of demyelination (distinguished via Fluoromyelin staining on the tetramethylrhodamine channel) were manually outlined in single-channel, grayscale images of lumbar spinal cord sections. Pixel area was measured, using ImageJ software (NIH), while blinded to the experimental groups. Percentage demyelination was calculated using the following formula: *100 – ([pixel area myelination/pixel area total white matter] × 100)*.

### Electrophysiology

Electrophysiological recordings were performed using a clinical electrodiagnostic system (Cadwell). During recordings mice were anesthetized using ketamine/xylazine anesthesia. A petroleum-based eye lubricant (Dechra) was applied to prevent corneal irritation and dryness. A thermostatically controlled, far-infrared heating pad was used to maintain body temperature (Kent Scientific). Hair from the right hind limb was removed with clippers (Remington, model VPG 6530) to allow for adequate electrode-skin contact. During anesthetized recordings, mice were placed in the prone position, and bilateral hind limbs were extended and affixed to the heating pad with Transpore medical tape (3M). Compound muscle action potential (CMAP) and single motor unit potential (SMUP) amplitudes were recorded from the right gastrocnemius muscle similar to our prior studies in aged mice (45, 46). Briefly, a pair of recording ring electrodes (Alpine Biomed) were placed at the proximal gastrocnemius (G1) and at the midtarsal region of the hind paw (G2). The skin underneath the ring electrodes was coated with electrode gel (Parker Laboratories) to reduce skin impedance. A disposable surface disc electrode was placed on the surface of the skin of the tail (Natus Neurology) as the common reference electrode (G0). Two 28-gauge monopolar electrodes (Natus Neurology) were inserted subcutaneously at the proximal right thigh and used as the cathode and anode to stimulate the sciatic nerve. A constant current stimulator was used to deliver pulses (0–10 mA current, 0.1 ms duration). Supramaximal stimulation was delivered to record the maximum CMAP amplitude. Then the stimulus was reduced, and a gradually increasing stimulus was used to elicit a total of 10 all-or-none CMAP increments. The 10 incremental responses were averaged to determine the average SMUP amplitude, which was used to calculate MUNE = maximum CMAP/average SMUP. To determine CMAP response following spinal cord conduction, the stimulating electrodes were placed subcutaneously at the base of the skull on each side of the spinal column. The spinal cord was stimulated using a constant current stimulator (0–40 mA, 0.2 ms) to elicit the maximum cervical motor evoked potential amplitude. During all recordings, high- and low-frequency filter settings were set at 10 kHz and 10 Hz, respectively. Peak-to-peak amplitudes were used for all analyses.

### Flow cytometry

For surface staining, cells were resuspended in 1× PBS with 2% fetal bovine serum, fixable viability dye (eFluor506, eBioscience), and anti-CD16/32 (clone 2.4G2, hybridoma, ATCC). Cells were then labeled with fluorescently labeled monoclonal antibodies specific for individual markers. For intracellular cytokine staining, cells were stimulated with PMA (50 ng/mL), ionomycin (2 μg/mL), and Brefeldin A (5 μg/mL) for 4–6 hours. Cells were then fixed and permeabilized with the Fixation/Permeabilization Kit (BD Biosciences) according to the manufacturer’s protocols and incubated with fluorescently labeled monoclonal antibodies. Data were acquired using a FACSMelody flow cytometer (BD Biosciences) and analyzed with FlowJo software (Tree Star).

### Antibodies

The following antibodies were obtained from eBioscience: CD11b (M1/70)-APC-eFluor 780, CD11c (N418)-PerCP-Cyanine5.5, CD45.1 (A20)-FITC and PE, CD45R/B220 (RA3-6B2)-PE, and Ki-67 (SolA15)-PE-Cyanine7. The following antibodies were obtained from Invitrogen: CD45 (30-F11)-eFluor 450 and GM-CSF (MP1-22E9)-FITC. The following antibodies were obtained from BD Pharmingen: Ly6G (1A8)-PE-Cy7. The following antibodies were obtained from BioLegend: CD4 (RM4-5)-APC. For in vivo GM-CSF blocking experiments, 500 μg of anti–mouse GM-CSF (MP1-22E9) or rat IgG2a isotype control (2A3) (BioXCell) was administered via i.p. injection.

### Multiplex cytokine assay

Cytokine levels were measured in spinal cord homogenates by Luminex multiplex bead-based analysis (MILLIPLEX MAP Mouse Cytokine/Chemokine Magnetic Bead Panel; MilliporeSigma) using the Bio-Plex 200 system (Bio-Rad Laboratories) according to the manufacturer’s protocols.

### Generation of BM chimeras

Femur, tibia, and humerus bones were harvested from donor mice. Marrow was flushed from the bone, then passed through a 70 μm mesh filter to generate a single-cell suspension, and red blood cells were lysed by a brief incubation in ACK lysis buffer (Quality Biological). Hosts were lethally irradiated with 2 doses of 6.5 Gy and reconstituted by tail vein injection of 2 × 10^6^ to 4 × 10^6^ donor BM cells. Recipient mice were allowed to reconstitute their circulating leukocyte pools for 6 weeks prior to use.

### Schematic design

All figure schematics were created with BioRender.com.

### scRNA-Seq analysis

#### Sequence mapping and preprocessing.

Mononuclear cells were isolated from 4 spinal cords per group and processed following the 10x Genomics Chromium Single Cell RNA v3 protocol. Libraries were sequenced on an Illumina NovaSeq instrument and counted with CellRanger v3.1.0 using mm10 reference genome GENCODE vM23/Ensembl 98. Data processing and visualizations of the scRNA-Seq data were performed using the Seurat package (v.4.0.4) in R (4.1.0). For the initial quality control filtering, we removed individual cells that detected fewer than 200 genes or more than 25,000 reads, as well as genes that were detected in fewer than 3 cells. We filtered outlier cells outside the range of 5 times median absolute deviation of that cell due to sequencing depth using scater in R. We also retained only cells with less than 10% mitochondrial reads and less than 50% ribosomal reads. Data were scaled to 10,000 transcripts per cell, then transformed to log space using Seurat’s LogNormalize method. The top 2000 highly variable genes in each sample were computed based on dispersion and mean.

#### Data integration and cell clustering.

To correct batch effects, anchor genes were identified from all samples using the FindIntegrationAnchors function in Seurat on the first 20 dimensions, and data were integrated via the Seurat IntegrateData function. Principal component analysis was performed on the top highly variable genes. The top 20 PCs were used to build a k-nearest-neighbors cell-cell graph with k = 30 neighbors. Cell clusters were identified using the Louvain graph-clustering algorithm with a resolution set to 0.4. We assigned the cell type labels for each cell cluster using small sets of known marker genes and cluster-specific genes. Finally, the data set was projected onto 2-dimensional space using UMAP dimensionality reduction with default parameters.

#### Differentially expressed gene analysis and enrichment test.

We identified cluster-specific genes for the clusters using the FindAllMarkers function, comparing the gene expression levels in a given cluster with the rest of the cells. The significance of difference was determined using a Wilcoxon rank-sum test with Bonferroni’s correction. Differentially expressed genes are determined by setting a threshold of the adjusted *P* value of less than 0.05 and absolute log_2_-fold change more than 0.25. We computationally selected individual cell types for between-group differential gene expression analysis. Note that the batch-corrected integration data were used only for cell clustering and dimensional reduction, and the differential gene expression analysis was performed using the normalized RNA assay slot in Seurat. Pathway enrichment analysis was performed using Enrichr using libraries including GO_2018 and KEGG_2019_MOUSE. We also used clusterProfiler’s universal enrichment analyzer to analyze our manually selected GO pathways and overlapping DEGs with public data sets. Volcano plots were generated for DEG visualization using EnhancedVolcano. Heatmaps were generated using ComplexHeatmap on *z* score–transformed normalized gene expression values.

#### Data availability.

RNA-Seq data are available in the NCBI’s Gene Expression Omnibus database under the following accession number: GSE200901.

### Bulk RNA-Seq analysis

Individual FASTQ files were trimmed for adapter sequences and filtered using fastp v0.20.0. Mouse reference genome GRCm38.p6 and gene annotation described by Gene Transfer Format were downloaded from Ensembl release 99 (January 2020). Reads alignment was performed against the reference genome using HISAT2 v2.1.0. Gene expression values for genes were quantified using the featureCounts tool of the Subread package v1.5.0-p2 in the unstranded mode. Genes detected in fewer than 10 total counts in at least 3 samples were removed from downstream analysis. The fold change was calculated using counts per million–normalized values using EdgeR.

### Statistics

Statistical analysis was performed in GraphPad Prism using paired or unpaired 2-tailed Student’s *t* test, or 1-way or 2-way ANOVA with correction for multiple comparisons, as indicated in the legends. Disease curves were compared by unpaired 2-tailed Student’s *t* tests with a Welch correction at individual days where indicated. Comparison for incidence, survival, and remission curves were performed using a log-rank test. Outliers were identified by ROUT analysis and removed as necessary. A *P* value less than 0.05 (*) was considered significant; **P < 0.01, ***P < 0.001, and ****P < 0.0001. A description of the statistical analysis used in RNA-Seq analysis is provided in the previous subsection.

### Study approval

All animal experiments were performed in accordance with an IACUC-approved protocol at The Ohio State University.

## Author contributions

JRA, ADJ, ARS, A Munie, and WDA performed experiments and data analysis. A Ma and CW oversaw RNA-Seq analysis. BMS and JRA wrote the manuscript and coedited it with the help of the other authors. BMS and JRA directed the studies.

## Supplementary Material

Supplemental data

## Version 1 05/05/2022

In-Press Preview

## Version 2 06/22/2022

Electronic publication
