# Supplementary material for: Biological aging of CNS-resident cells alters the clinical course and immunopathology of autoimmune demyelinating disease
Source: JCI Insight. 2022 Jun 22;7(12):e158153. doi: 10.1172/jci.insight.158153 (PMC9309055; doi:10.1172/jci.insight.158153)
Supplement: Supplemental data [file jciinsight-7-158153-s125.pdf]

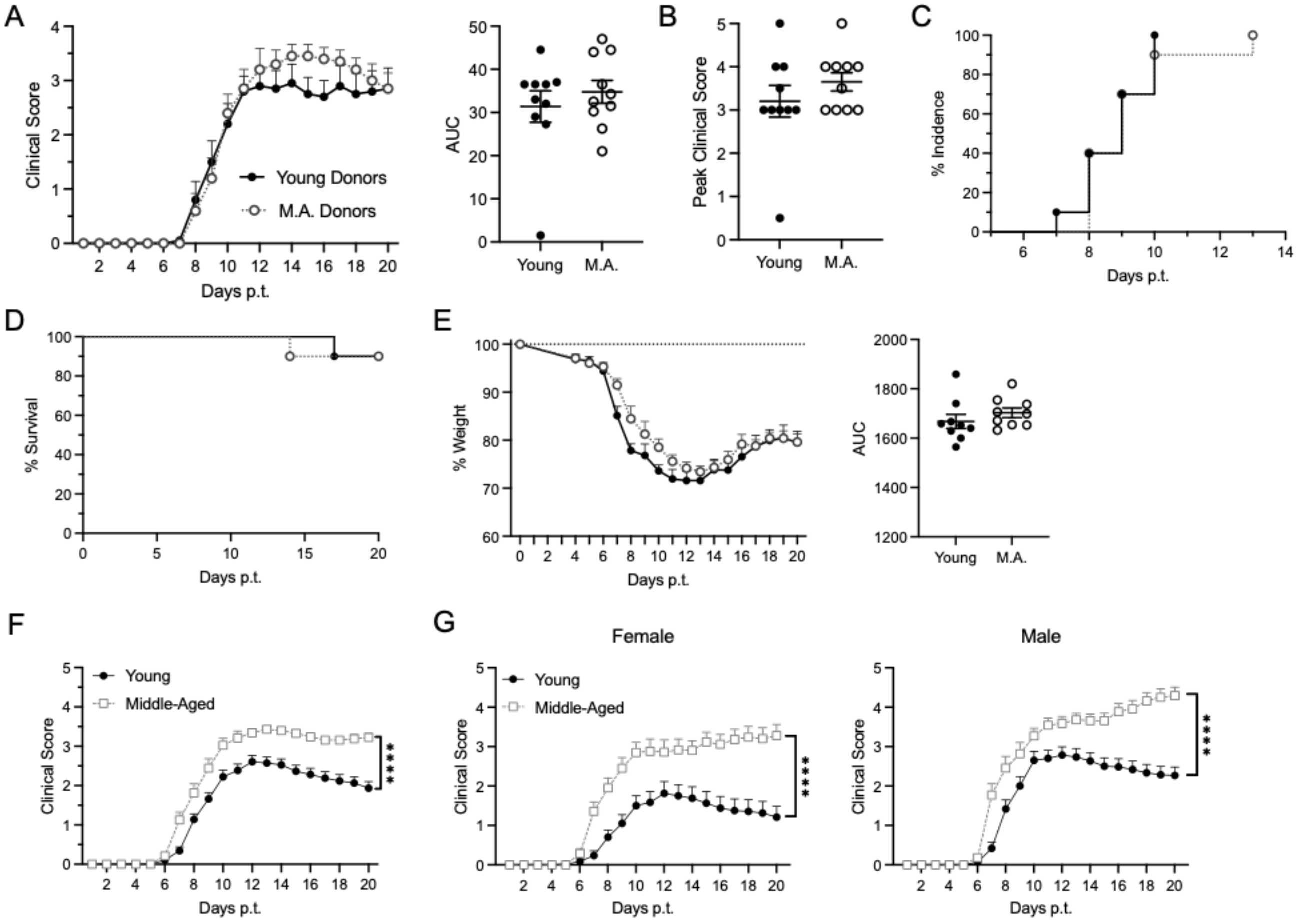

Supplemental Figure 1. The age of MOG-primed donor CD4+ T cells does not impact the clinical course of adoptively transferred EAE. MOG/CFA primed lymph node cells from young adult (8-12 week old) or middle-age (M.A., 40-44 week old) mice were cultured with MOG_{35-55} peptide and Th17 polarizing factors. The cells were harvested 96 hours later. CD4+ T cells were purified and injected into naïve, syngeneic young adult mice (n=10 mice/ group). (**A**) Mean clinical scores of mice in each group (left) and areas under the curve (AUC) of individual mice (right). (**B**) Peak clinical scores for individual mice. (**C**) Disease incidence in each group over time. (**E**) Percent of surviving mice in each group over time. (**E**) Mean change in weight (shown as percent of baseline weight) of mice in each group throughout the clinical course (left), and AUCs of individual mice (right). (**F**) Clinical courses of young (n=70) and middle-aged (n=43) adoptive transfer recipients that survived throughout the duration of the study. Data are pooled from 4 independent experiments. (**G**) Clinical courses of young and middle-aged adoptive transfer recipients, separated by sex. n=23-35/ group. Data were pooled from 2 independent experiments. Curves in **F** and **G** were compared using a mixed effects model. Error bars indicate mean ± SEM. ****p <0.0001.

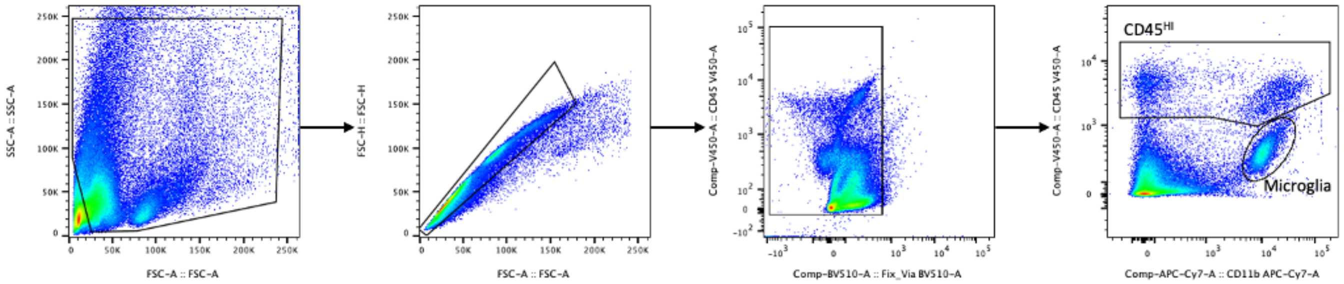

Supplemental Figure 2. Representative gating strategy for flow cytometric analysis and FACS isolation. Single cells were gated via forward scatter (FSC) and side scatter (SSC), and dead cells were excluded using a fixable viability dye. Microglia were identified as $CD45^{int}CD11b^{+}$, infiltrating myeloid cells as $CD45^{hi}CD11b^{+}$, and infiltrating lymphocytes as $CD45^{hi}CD11b^{-}$.

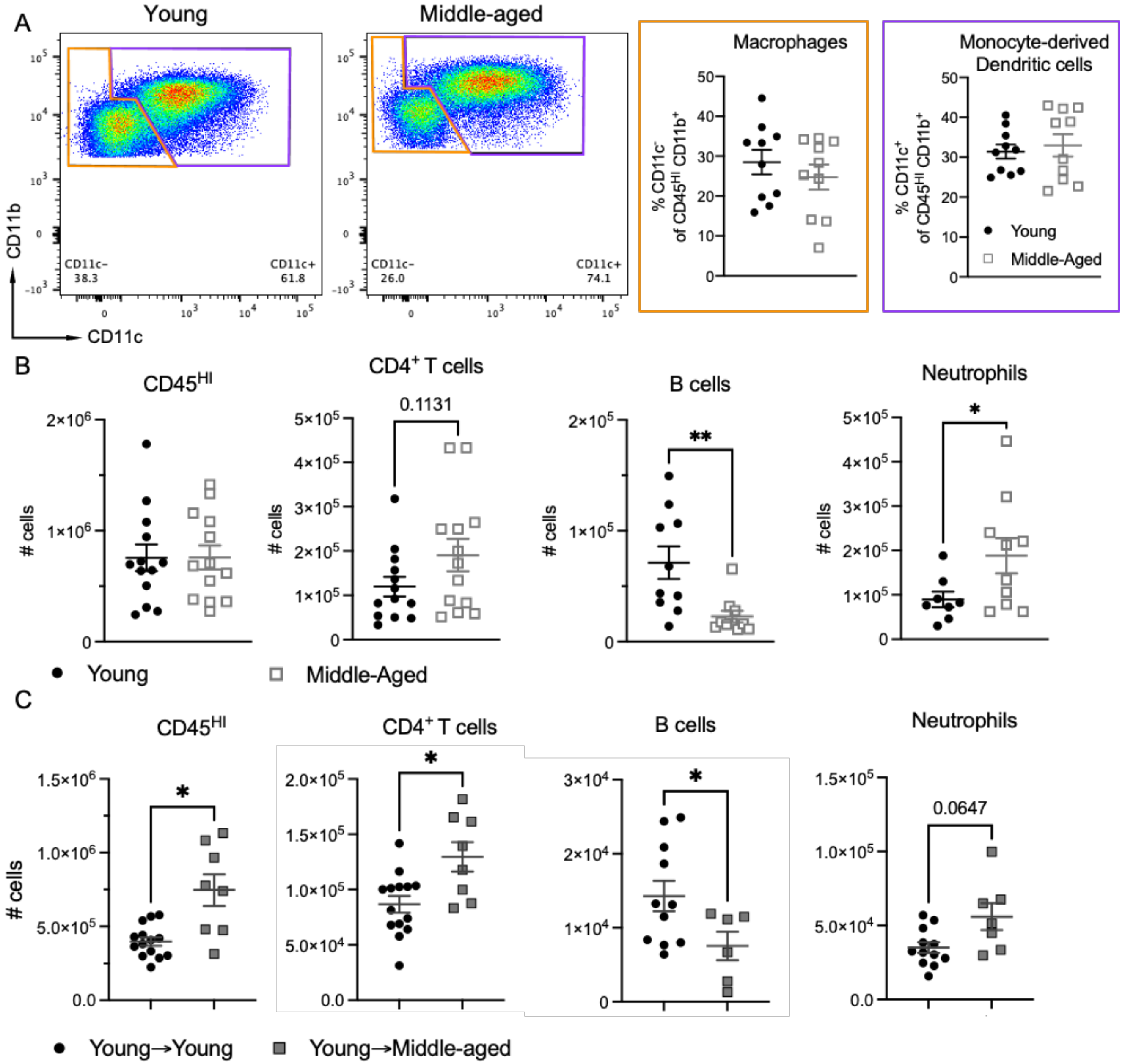

Supplemental Figure 3. The frequencies of monocyte-derived dendritic cells and macrophages and total number of CNS infiltrates of young and middle-aged adoptive transfer recipients, and chimeric mice, at peak EAE. CNS inflammatory cells were harvested from the spinal cords of young and middle-aged mice at day 10 adoptive post-transfer. (**A**) Representative dot plots of CNS infiltrating myeloid cells (left panels). Frequencies of macrophages (CD11b^{+} Ly6G^{-} CD11c^{-}) and monocyte-derived dendritic cells (CD11b^{+} Ly6G^{-} CD11c^{+}) among CD45^{HI} mononuclear cells isolated from the spinal cords of individual adoptive transfer recipients (right panels). (**B, C**) Total numbers of CD45^{HI} infiltrating immune cells, CD4^{+} T cells, donor (CD45.1^{+}) CD4^{+} T cells, B cells, and neutrophils per spinal cord harvested from non-chimeric mice (**B**), and young→young and young→middle-aged bone marrow chimeras (**C**), on day 10 postTh17 cell transfer. Each symbol represents a data point generated from a single mouse. Statistical significance was determined using the unpaired 2-tailed Student's *t* test. *p <0.05, **p <0.01.

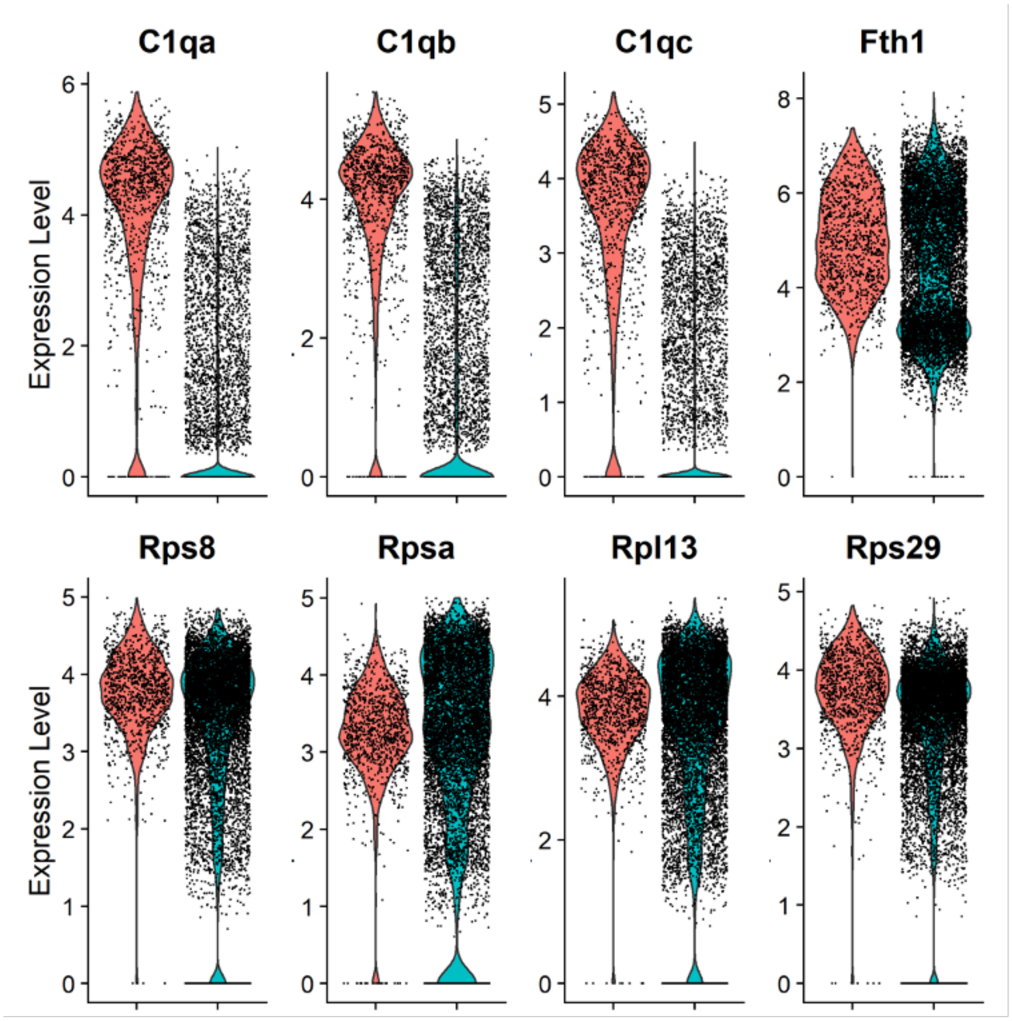

Supplemental Figure 4. Microglia from middle-aged mice with EAE express high levels of genes identified in microglia located in the rims of chronic active pMS lesions. CD45$^+$ mononuclear cells were FACS sorted from middle-aged mice with EAE on day 10 post-cell transfer and subjected to scRNAseq. Selected differentially expressed genes in microglia (red) compared with all other cells (blue), shown as violin pots. Numbers on the y-axis correspond to the z score for each gene.
